# Novel Cellulose Acetate-Based Monophasic Hybrid Membranes for Improved Blood Purification Devices: Characterization under Dynamic Conditions

**DOI:** 10.3390/membranes11110825

**Published:** 2021-10-27

**Authors:** Adriana Janeca, Flávia S. C. Rodrigues, Maria Clara Gonçalves, Mónica Faria

**Affiliations:** 1 Departamento de Engenharia Química, Instituto Superior Técnico, Universidade de Lisboa, Av. Rovisco Pais, 1049-001 Lisboa, Portugal; adriana.janeca@tecnico.ulisboa.pt (A.J.); flavia.rodrigues@tecnico.ulisboa.pt (F.S.C.R.); clara.goncalves@tecnico.ulisboa.pt (M.C.G.); 2 CeFEMA, Center of Physics and Engineering of Advanced Materials, Av. Rovisco Pais, 1049-001 Lisboa, Portugal; 3 CQE, Centro de Química Estrutural, Av. Rovisco Pais, 1049-001 Lisboa, Portugal

**Keywords:** monophasic hybrid membrane, sol-gel, phase inversion, blood purification, ultrafiltration, crossflow filtration, convection, hemodialysis

## Abstract

A novel cellulose acetate-based monophasic hybrid skinned amine-functionalized CA-SiO_2_-(CH_2_)_3_NH_2_ membrane was synthesized using an innovative method which combines the phase inversion and sol-gel techniques. Morphological characterization was performed by scanning electron microscopy (SEM), and the chemical composition was analyzed by Fourier transform infrared spectroscopy in attenuated total reflection mode (ATR-FTIR). The characterization of the monophasic hybrid CA-SiO_2_-(CH_2_)_3_NH_2_ membrane in terms of permeation properties was carried out in an in-house-built single hemodialysis membrane module (SHDMM) under dynamic conditions. Permeation experiments were performed to determine the hydraulic permeability (Lp), molecular weight cut-off (MWCO) and the rejection coefficients to urea, creatinine, uric acid, and albumin. SEM confirmed the existence of a very thin (<1 µm) top dense layer and a much thicker bottom porous surface, and ATR-FTIR showed the main bands belonging to the CA-based membranes. Permeation studies revealed that the Lp and MWCO of the CA-SiO_2_-(CH_2_)_3_NH_2_ membrane were 66.61 kg·h^−1^·m^−2^·bar^−1^ and 24.5 kDa, respectively, and that the Lp was 1.8 times higher compared to a pure CA membrane. Furthermore, the CA-SiO_2_-(CH_2_)_3_NH_2_ membrane fully permeated urea, creatinine, and uric acid while completely retaining albumin. Long-term filtration studies of albumin solutions indicated that fouling does not occur at the surface of the CA-SiO_2_-(CH_2_)_3_NH_2_ membrane.

## 1. Introduction

Chronic kidney disease, a growing public health concern affecting 11–13% of the global population [1], is defined by an irreversible worsening of renal function which can lead to end-stage renal disease (ESRD). Currently, about 3 million ESRD patients receive renal replacement therapies to survive, and the number is likely to reach 10 million by 2030 [1]. The progressive loss of kidney function is linked to the retention of metabolic waste products, also known as retention solutes or uremic toxins (UTs), which are normally excreted by healthy kidneys. UTs are classified based on their molecular weight (MW), removal pattern during hemodialysis (HD), and relative affinity for protein binding, and are divided into three major groups: (1) small water-soluble compounds (MW < 500 Da); (2) middle molecules (MW ≥ 500 Da); and (3) protein-bound uremic toxins (PBUTs; MW < 500 Da when free, >66.5 kDa when bound to albumin (MW 66.5 kDa)) [2].

Hemodialysis is the renal replacement therapy most widely used to purify the blood of ESRD patients. The hemodialyzer is the key component of the extracorporeal blood circulation circuit, as it is responsible for removing accumulated UTs and water while simultaneously retaining vital blood components, such as proteins, through a bundle of semi-permeable membranes [3]. The main mass separation mechanisms used to remove water and UTs from the blood of ESRD patients through semi-permeable membranes are diffusion, convection, and adsorption.

Although different classification schemes for HD membranes have been proposed [4], they are traditionally classified according to water flux—a term also referred to as water permeability. The clinical parameter used to characterize the water permeability of a dialyzer is the ultrafiltration coefficient, K_UF_, expressed in mL/h/mmHg units. The water permeability of a hemodialyzer is found by in vitro ultrafiltration experiments, in which bovine blood is ultrafiltered at varying transmembrane pressures (TMPs). At low TMPs, the ultrafiltration rate (Q_UF_) varies linearly with TMP and then reaches a plateau at relatively high TMP values [5]. The slope of the linear portion of the Q_UF_ versus the TMP curve defines the dialyzer K_UF_. The membrane pore size is the membrane property with the largest impact on water permeability, whereas the ultrafiltrate flux (J_UF_) is approximately proportional to the fourth power of the mean membrane pore radius [6]. As such, small changes in pore size have an exceptionally large effect on water permeability. K_UF_ is the most-used parameter for classification purposes; according to the United States Food and Drug Administration, a value of 12 mL/h/mmHg differentiates low-permeability and high-permeability dialyzers [7].

Traditionally, dialysis membranes have been broadly classified on the basis of their composition as cellulosic or synthetic [8]. The earliest hemodialysis membranes consisted of regenerated cellulose, which rendered small pore sizes—enabling the clearance of molecules smaller than 5000 Daltons into the dialysate, and were associated with an inflammatory response [8]. In the early 1970s, in an attempt to enhance the hemocompatibility of regenerated cellulose membranes, researchers turned to modified cellulose materials, such as cellulose acetate (CA) and cellulose triacetate, where a large percentage of the hydroxyl groups is replaced with an acetate radical, eliminating the active surface sites for complement protein interaction. This modification also led to the improvement of solute mass transfer [9]. This decade also witnessed the development of synthetic membranes, such as polyacrylonitrile (PAN) and polysulfone (PS) [6]. These latter membranes differed fundamentally from cellulosic materials not only in their polymeric composition, but also in a number of other features including larger pore size, decreased thickness, and hydrophobicity.

Due to their hydrophobicity, synthetic HD membranes must be rendered hydrophilic before they can be used in HD, thus adding an extra step to the preparation process. Furthermore, their final hemocompatibility and filtration performance depends on the hydrophilization processes they are subjected to [10]. In terms of pore size, they are generally larger in synthetic membranes than in cellulosic membranes [8], allowing for a faster extraction of water and potential removal of middle-sized and protein-bound retention solutes [11].

The evolution of biomaterials and improved membrane synthesis technology during the 1990s resulted in new cellulosic and synthetic membranes with specific characteristics and refined properties. The development of integral asymmetric CA membranes [12] by means of the wet-phase inversion technique paved the way to the synthesis of integrally skinned asymmetric membranes with a multitude of structures covering a wide range of membrane processes, from ultrafiltration (UF) [13] to reverse osmosis [14]. CA membranes were used to develop low-flux hemodialyzers [15], high-flux hemodialyzers [16] and even low-flux hemodialyzers with efficient β_2_-microglobulin (β2m) removal [17]—a marker for middle-sized molecules.

Due to the higher water permeability and efficiency in the removal of UTs of larger molecular weight, the majority of HD membranes on the market today are made from synthetic polymers [18]. Nevertheless, the highly porous structure of synthetic membranes has disadvantages: studies focused on the dialysate/ultrafiltrate removed from patients subjected to high-flux HD identified the convective removal of useful and even vital compounds found in blood [11,18], such as albumin and vitamin D-transporting protein [19]. At a much smaller scale, melt-spun CA membranes are being successfully used in HD [20,21], with studies reporting their low toxicity and reduced complement activation [22].

Cellulose is the most abundant and available biorenewable polymer on Earth’s crust. Furthermore, the long chain biopolymeric carbohydrate molecules primarily composed of monosaccharide units (which compose cellulosic materials) can be easily functionalized, making it a versatile material with immense potential in biomedical applications. Nevertheless, CA membranes exhibit some drawbacks—namely, limited chemical resistance, low mechanical strength, low shelf life, and small pore size [23]. To overcome these limitations, our research group has focused on the development of novel monophasic hybrid CA-based membranes, which combine the high mechanical and thermal stability of the inorganic material with the flexibility, ductility, and processability of CA [24,25,26,27,28]. In a recent study [27], a modified version of the phase inversion technique [13] coupled with the sol-gel method [29,30] was used to synthesize novel monophasic hybrid skinned amine-functionalized CA-SiO_2_-(CH_2_)_3_NH_2_ membranes.

It is envisioned that the incorporation of NH_2_ groups into cellulosic membranes may translate into preferential interactions with proteins present in the blood—especially albumin (that may or may not be bound to PBUTs). The monophasic hybrid membranes are fabricated under acidic conditions, giving rise to the protonation of NH_2_ groups. Therefore, when the hybrid membranes are in close contact with albumin, a strong electrostatic interaction between the positively charged $-{\mathrm{NH}}_{3}^{+}$ group (from the hybrid membrane) and the −COOH or −NH (from albumin) occurs. This electrostatic interaction may allow the displacement of PBUTs from albumin.

The mechanical properties of the CA-SiO_2_-(CH_2_)_3_NH_2_ membranes were enhanced and permeation studies revealed that the introduction of propyl-amine groups increased the hydraulic permeability by a factor of three when compared to pure CA membranes [27].

The aim of this study is to evaluate the potential of monophasic hybrid CA-SiO_2_-(CH_2_)_3_NH_2_ membranes for application in blood purification devices such as HD, where the preferential permeation of uremic toxins and the retention of vital blood components such as albumin are a mandate. For this, a monophasic hybrid CA-SiO_2_-(CH_2_)_3_NH_2_ membrane containing 95 wt% CA and 5 wt% SiO_2_ + SiO_2_-(CH_2_)_3_NH_2_ was synthesized and characterized in an in-house-built single hemodialysis membrane module (SHDMM) under dynamic conditions. For comparison purposes, a pure CA membrane was also synthesized and characterized. The surface and cross-section morphologies of the membranes were characterized by scanning electron microscopy (SEM) and the chemical composition was analyzed by Fourier transform infrared spectroscopy in attenuated total reflection mode (ATR-FTIR). Permeation experiments were carried out to evaluate the membranes’ performance in terms of hydraulic permeability (Lp), molecular weight cut-off (MWCO), and rejection coefficients to a group of small water-soluble compounds and bovine serum albumin (BSA).

## 2. Materials and Methods

### 2.1. Materials

Membranes were synthesized with cellulose acetate from Sigma-Aldrich (Steinheim, Germany) (CA; (C_6_H_7_O_2_(OH)_3_, ~30,000 g/mol, reagent grade ≥ 97%, esterification degree ~40%), tetraethyl orthosilicate (TEOS; Si(OC_2_H_5_)_4_, 208.33 g/mol, reagent grade 98%), purchased from Alfa Aesar (Karlsruhe, Germany), 3-(triethoxysilyl)–propylamine (APTES; C_9_H_23_NO_3_Si, 221.37 g/mol, reagent grade ≥ 98%) purchased from Sigma-Aldrich (Steinheim, Germany), formamide (CH_3_NO, 45.02 g/mol, ≥99.5%) purchased from Carlo Erba (Val-de-Reuil, France), acetone (C_3_H_6_O, 58.08 g/mol, ≥99.6%) purchased from Labsolve (Zedelgem, Belgium), and nitric acid (HNO_3_, 63.01 g/mol, 1.39 g/mL at 20 °C, 65% *v*/*v*) purchased from LabSolve (Zedelgem, Belgium).

Membrane drying was performed with isopropanol (≥99.8%) from Honeywell (Seelze, Germany) and n-hexane (≥95%) from Carlo Erba (Val-de-Reuil, France).

Permeation experiments were carried out with urea (MW 60.06 g/mol) purchased from Merck (Darmstadt, Germany), creatinine (MW 113.12 g/mol) purchased from Sigma-Aldrich (Steinheim, Germany), uric acid (MW 168.11 g/mol) purchased from Alfa Aesar (Kandel, Germany) and bovine serum albumin (BSA; MW 66.5 g/mol) purchased from Sigma-Aldrich (Steinheim, Germany).

MWCO was studied using polyethylene glycol (PEG) 400 (MW 400 g/mol) purchased from Sigma-Aldrich (Steinheim, Germany), PEG 3000 (MW 3000 g/mol), PEG 6000 (MW 6000 g/mol), PEG 10,000 (MW 10,000 g/mol) purchased from Merck (Hohenbrunn, Germany), PEG 20,000 (MW 20,000 g/mol) purchased from Sigma-Aldrich (Steinheim, Germany) and PEG 35,000 (MW 35,000 g/mol) purchased from Merck (Hohenbrunn, Germany).

To dissolve BSA, phosphate buffer saline (PBS) was prepared using sodium chloride (NaCl, 58.44 g/mol, ≥99.5%) purchased from Merck (Darmstadt, Germany), potassium chloride (KCl, 74.56 g/mol, ≥99.5%) purchased from Panreac (Barcelona, Spain), potassium dihydrogen phosphate (KH_2_PO_4_, 136.09 g/mol, ≥99.5%) purchased from Merck (Darmstadt, Germany) and disodium hydrogen phosphate dihydrate (Na_2_HPO_4_·2H_2_O, 177.99 g/mol, ≥99.5%) purchased from Merck (Darmstadt, Germany).

The quantification of BSA was carried out according to the Bradford protein assay. Bradford reagent was prepared using Coomassie Brilliant Blue G-250 (C_47_H_48_N_3_NaO_7_S_2_, 854.04 g/mol) purchased from Panreac (Barcelona, Spain), ethanol (C_2_H_6_O, 46.07 g/mol, 96% *v/v*) purchased from Manuel Vieira & Cª (Torres Novas, Portugal) and phosphoric acid (H_3_PO_4_, 98.00 g/mol, 1.71 g/mL at 20 °C, reagent grade 85%) purchased from Honeywell (Seelze, Germany).

All chemicals used in the synthesis, drying and characterization of the monophasic membranes were used without further purification.

### 2.2. Membrane Fabrication

The monophasic hybrid integrally skinned asymmetric CA-SiO_2_-(CH_2_)_3_NH_2_ membrane was prepared by coupling the phase inversion method with the sol-gel technique.

The casting solution of the monophasic hybrid CA-SiO_2_-(CH_2_)_3_NH_2_ membrane was prepared in two steps. First, 16.4 g of cellulose acetate, 29.0 g of formamide and 51.1 g of acetone were mixed in a reaction vessel to allow the complete dissolution of cellulose acetate. After 5 h of mixing, 2.4 g of TEOS, 0.6 g of APTES (to uphold amine in situ functionalization) and 12 drops of nitric acid were added to the mixture, promoting hydrolysis and hetero-condensation during the casting solution homogenization step. The final solution was placed on an agitation plate for another 19 h, resulting in a total homogenization time of 24 h for CA.

A reference casting solution of the pure CA membrane was prepared by mixing 17.0 g of cellulose acetate, 30.0 g of formamide and 53.0 g of acetone for 24 h.

The pure CA and CA-SiO_2_-(CH_2_)_3_NH_2_ casting solutions were cast on glass plates with a 250 μm Gardner knife at room temperature and a solvent evaporation time of 30 s, after which they were quenched into a gelation bath containing water at a temperature of 4 ± 1 °C, rendering the pure CA and monophasic hybrid CA-SiO_2_-(CH_2_)_3_NH_2_ membranes. After a residence time of approximately 3 h in the coagulation bath, the CA and CA-SiO_2_-(CH_2_)_3_NH_2_ membranes were detached from the glass plate and stored separately in deionized water at 4 ± 1 °C.

The sol-gel reactions which occur during the hybrid membranes’ casting solution step have been previously described [27]. Briefly, after the sol-gel hydrolysis reactions, the silanol groups from the inorganic (TEOS) and hybrid (APTES) monomers hetero-condensate with the C–OH groups from the CA polymer, forming a new Si–O–C covalent bond. The sol-gel process was developed under acid catalysis. The new hybrid casting solution is then cast to form a monophasic hybrid membrane, based on carbon, silica, and amine chemical species, originating complex carbon-silica networks.

### 2.3. Membrane Characterization

#### 2.3.1. Scanning Electron Microscopy (SEM)

Prior to being imaged by scanning electron microscopy (SEM), the CA and CA-SiO_2_-(CH_2_)_3_NH_2_ membranes were submitted to the drying process described by Lui et al. [31]. Samples of both membranes were then fractured in liquid nitrogen, mounted on a stub and sputter-coated with gold. Micrographs of the top dense surface (magnification: 1000×), cross-sections (magnification: 700×) and the porous bottom layer (magnification: 4000×) were obtained. The active layer and total thickness of the membranes were calculated using ImageJ software (version 1.53 k, from National Institutes of Health). For each membrane, three randomly selected zones from the images depicting the cross section of the CA and CA-SiO_2_-(CH_2_)_3_NH_2_ membranes were measured, and the mean thickness and standard deviation of the entire membrane and active layer were calculated. In order to determine the average pore size of the porous bottom layer, the images were binarized using a threshold adjustment and subsequently analyzed via the Measure tool of the ImageJ software.

#### 2.3.2. Attenuated Total Reflection-Fourier Transform Infrared Spectroscopy (ATR-FTIR)

Attenuated total reflection-Fourier transform infrared spectroscopy (ATR-FTIR) was used to analyze the chemical composition of the active layer surfaces of dried CA and CA-SiO_2_-(CH_2_)_3_NH_2_ membranes. Infrared spectra of the samples with the active layer facing upwards were obtained with a PerkinElmer Frontier FT-IR spectrometer (940 Winter Street, Waltham, MA 02451, USA), using a Pike Miracle Single Reflection ATR sampling accessory from Pike Technologies, with a Ge crystal (Graseby Specac, Smyrna; sampling depth: 0.2–1.1 μm at 4000–650 cm^−1^). Each spectrum was obtained by averaging 256 scans with a resolution of 4 cm^−1^. The infrared spectra were reported as transmittance versus wavenumber.

### 2.4. Experimental Setup

#### 2.4.1. Single Hemodialysis Membrane Module (SHDMM)

**Figure 1:**
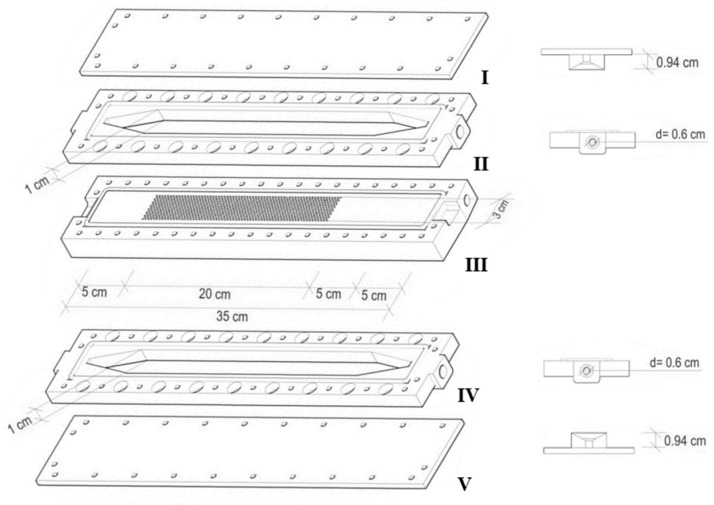
Schematic representation of the single hemodialysis membrane module. Unit I seals the feed flow chamber, represented by Unit II, while Unit V seals the permeate collecting chamber, represented by Unit IV. Unit III is the supporting surface for the membrane to be tested.

A single hemodialysis membrane module was custom-made by micromachining of acrylic plates. Figure 1 shows the schematic representation and dimensions of the five units which compose the SHDMM. Unit II defines the feed flow chamber, while the purpose of the perforated piece, Unit III, is to support the membrane. Unit IV defines the chamber in which the permeate is collected and Units I and V seal the top feed flow and bottom permeate collecting chambers, respectively. The membrane is placed with its active layer facing upwards on top of Unit III, with a filter paper beneath it to prevent damage to the membrane when all units are sealed together by using stainless-steel screws. Because of the large thickness of Unit III (1 cm), on top of which the membrane is placed, the solutions in the feed flow channel and the permeate compartment do not interact closely enough to establish a concentration gradient. Therefore, the solute diffusion between the compartments is negligible and it is assumed that fluid and solute removal occur exclusively by convection. The feed flow and permeate collecting chambers were designed to be slit-like microchannels, where the channel height (2B) is much smaller than the other two dimensions: width (W) and length (L). As such, the dimensions of the microchannels are 2B = 0.3 cm, W = 3.0 cm, and H = 25.0 cm (2B ≪ W and L). The effective membrane surface area is defined by the area of the membrane clamped between the O-ring which seals Units I and II and is 105 cm^2^.

#### 2.4.2. Feed Circulation Circuit

**Figure 2:**
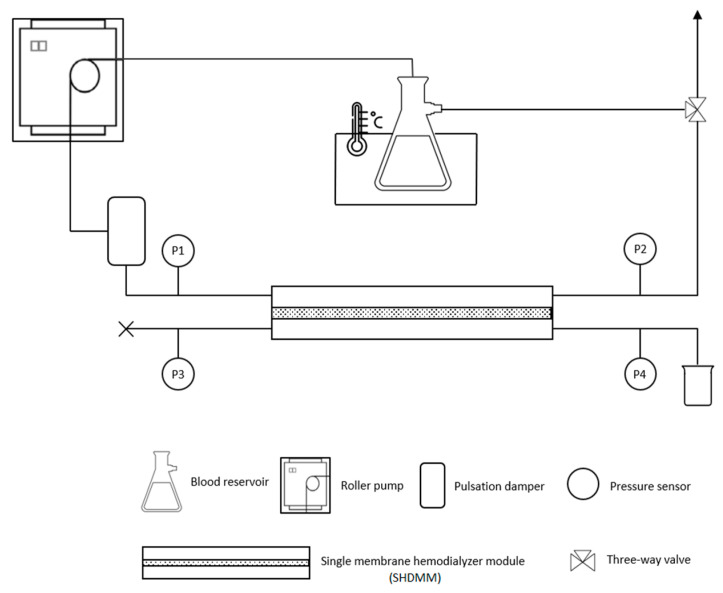
Schematic representation of the experimental setup used for permeation experiments under dynamic conditions.

Figure 2 represents the circuit which simulates the extracorporeal blood circulation circuit found in HD machines. The feed reservoir, a 500 mL glass Kitasato, is placed in a thermostatically controlled water bath (model CORIO C, from JULABO, Seelbach, Germany) to maintain the feed solution at the average normal body temperature, 37 °C. The feed solution is pumped from the reservoir and through the circuit by a peristaltic pump similar to the ones used in extracorporeal circulation such as dialysis (model ECOLINE VC-360, from ISMATEC, Wertheim, Germany), and its pulsation effect is attenuated by a damper, allowing the feed fluid to enter the SHDMM at a constant pressure. The ultrafiltrate is collected in the permeate collecting chamber, and the fraction of the feed solution which has not been permeated is recirculated back to the feed reservoir. A three-way valve placed midway between the outlet and the reservoir enables the collection of feed samples. Throughout the entire circuit, polyvinyl chloride (PVC) tubing (Tygon Saint-Gobain, La Defense, Courbevoie, France) with an inner diameter of 3 mm and a Shore hardness of 55 A was used to ensure that the shedding of particles from the interior of the tubing—a phenomenon known as spallation [32,33]—is low.

Four medical grade pressure sensors (Deltran^®^ Utah Medical Products, Inc., Midvale, UT, USA)—P1, P2, P3, and P4—register the pressure at crucial points of the circuit. P1 and P2 register the pressure at the inlet and outlet of the feed flow chamber, respectively, whereas P3 and P4 register the pressure at the inlet and outlet of the permeate collecting chamber, respectively. The pressure sensors are connected to a data acquisition card (National Instruments cDAQ-9172, USA) which records the pressure variations throughout time via a LabView module (National Instruments, 9237, Austin, TX, USA). The cross to the left of the sensor represented by P3 indicates that the tube is clamped, and therefore the ultrafiltrate is collected by the tube placed after P4.

#### 2.4.3. Characterization of the SHDMM: Pressure Profile, Pressure Drop, Transmembrane Pressure, Microchannel Height, Shear Rate and Shear Stress at the Wall

**Figure 3:**
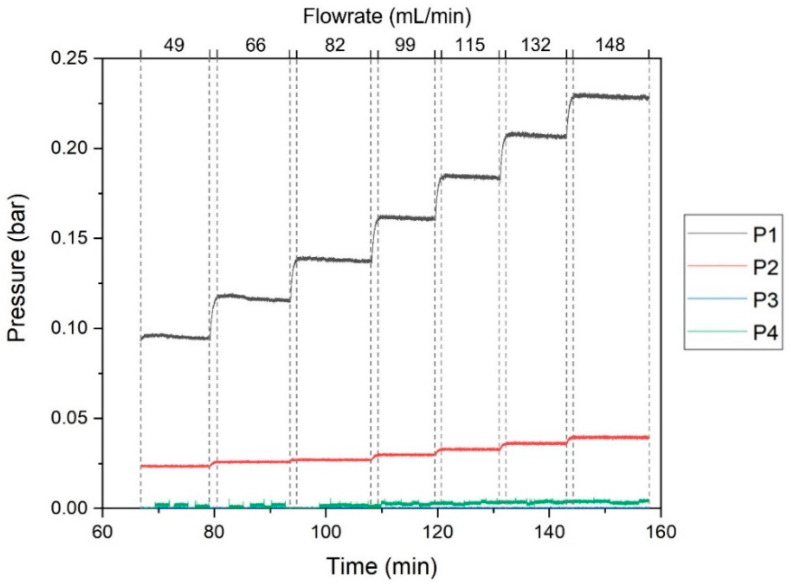
Pressure profile of the circuit during the circulation of DI water through the SHDMM circuit at different values of Q_F_, which are indicated delimited by the dashed lines. The pressure data is color-coded according to the different sensors: black line—P1, red line—P2, green line—P3, and dark blue line—P4.

To characterize the SHDMM, a permeation experiment was performed to obtain a typical pressure profile for the flow of deionized (DI) water at different feed volumetric flow rates (Q_F_) through the SHDMM: 49 mL/min, 66 mL/min, 82 mL/min, 99 mL/min, 115 mL/min, 132 mL/min, and 148 mL/min. Figure 3 shows the pressure profile of the system registered continuously throughout the experiment. Each plateau represents the pressure registered for each pressure sensor resulting from the Q_F_ imposed by the peristaltic pump.

The pressure drop (ΔP) across the feed flow channel is defined by:

ΔP = P_1_ − P_2_ (1)

The transmembrane pressure is the hydrostatic pressure difference between the feed flow chamber and permeate collecting compartment, acting as the driving force for solute removal, and has unique values along the length of the membrane [5]. Assuming a linear variation of fluid pressure with axial distance along the channel, TMP is defined by:

(2) $$\mathrm{TMP}=\frac{P_{1}\;{+\;P}_{2}}{2}-\frac{P_{3}{\;+\;P}_{4}}{2}$$

As mentioned in Section 2.4.1, the feed flow microchannel has a slit-like geometry where the channel height (2B = 0.3 cm) is much smaller than the other two dimensions: width (W = 3.0 cm) and length (L = 25.0 cm). When the membrane is placed inside the SHDMM, the height of the feed flow microchannel is no longer 0.3 cm and must be accurately calculated.

The half-height of the microchannel (B) is obtained by an equation analogous to the Hagen–Poiseuille law for circular tubes and describes the fully-developed laminar flow of a Newtonian fluid in a narrow slit [34]:

(3) $$B=\sqrt[{3}]{\frac{3}{2}\frac{\mu L}{W}\frac{Q_{F}}{\Delta P}}$$

where μ is the viscosity of the fluid, L is the length of the microchannel, Q_F_ is the feed flow rate, W is the width of the microchannel and ΔP is the pressure drop across the microchannel.

The shear stress exerted at the flow boundaries, or walls of the microchannel, τ, can be calculated by balancing the shear force at the wall against the pressure gradient for a slit channel [34]:

(4) $$\tau =\frac{{3\mu Q}_{F}}{{2B}^{2}W}$$

Shear rates, γ, at the wall are found by dividing the shear stress by the viscosity:

(5) $$\gamma =\frac{\tau }{\mu }=\frac{{3\mu Q}_{F}}{{2B}^{2}W}$$

Shear rates and shear stress in the device were varied from 4000 to 11,800 s^−1^, and from 3 to 8 Pa, respectively.

### 2.5. Permeation Experiments under Dynamic Conditions

#### 2.5.1. Water Permeability

Permeation experiments were performed in the circuit containing the SHDMM to characterize the monophasic hybrid CA-SiO_2_-(CH_2_)_3_NH_2_ and pure CA membranes in terms of pure water permeability.

Ultrafiltration flux, J, is defined as the amount of permeate produced per unit area of membrane surface per unit time:

(6) $$J=\frac{V}{A\;\times \;t}$$

where V is the volume of permeate, A is the membrane permeation area, and t is the measuring time.

The water permeability, L_P_, is obtained by the slope of the straight line obtained when J is represented as a function of TMP:

(7) $$L_{P}=\frac{J}{\mathrm{TMP}}$$

The range of the TMP used was 0.0591, 0.0708, 0.0819, 0.0943, 0.107, 0.120 and 0.132 bar (45, 53, 61, 71, 80, 90 and 100 mm Hg), corresponding to Q_F_ values of 49, 66, 82, 99, 115, 132, and 148 mL/min, respectively.

The feed temperature was kept at 37 °C, equivalent to the normal body temperature of a healthy adult, for all the permeation experiments. For comparison purposes with other membranes on the market, the water permeability found at 37 °C for the CA-SiO_2_-(CH_2_)_3_NH_2_ and pure CA membranes was standardized for a temperature of 25 °C by Equations (8)–(11) [35,36]:

(8) $$\;\ln\mu \;=\;-6.78\;+\;\frac{1983}{T}\;(r\;=\;0.99923)$$

Equation (8) can be written in terms of mass flux and assumes the following form, when solved for J_m_:

(9) $$J_{m}\;=\;\rho \frac{L_{P}}{\mu }\Delta P$$

As neither L_P_ nor ΔP are temperature-dependent, the previous expression can be rewritten as:

(10) $$\frac{J_{m}(T)\;\mu (T)\;}{\rho (T)}{=\;L}_{P}\Delta P$$

Ultimately, the relationship between two fluxes, J_m,1_ and J_m,2_, at different temperatures, T_1_ and T_2_, respectively, is given by:

(11) $$\frac{J_{m,1}\mu_{1}\;}{\rho_{1}}=\frac{J_{m,2}\mu_{2}\;}{\rho_{2}}$$

#### 2.5.2. Molecular Weight Cut-Off

The molecular weight cut-off (MWCO) of a membrane is defined as the molecular weight at which 90% of the solute is rejected by the membrane [37]. To determine the MWCO of each membrane, a retention curve was constructed by measuring the rejection coefficient, f, of various polyethylene glycols (PEGs) with increasing molecular weights (MWs) of 400 Da, 3000 Da, 6000 Da, 10,000 Da, 20,000 Da, and 35,000 Da, respectively. The rejection coefficient, R, is defined by:

(12) $$R=\frac{C_{F\;}{-\;C}_{P}\;}{C_{F}}$$

where C_P_ corresponds to the concentration of the permeate at t = 60 min and C_F_ corresponds to the initial feed solution concentration. The initial concentration of the feed solutions of each PEG was 485 ± 76 mg/L and the permeation studies were performed for 1 h at a Q_F_ of 100 ± 5 mL/min and a TMP of 0.1 bar (76 ± 1 mmHg). Solute concentrations were determined in the permeate, initial feed, and final feed samples using a total organic carbon analyzer (TOC-VCPH/CPN, Shimadzu, Japan).

For the CA-SiO_2_-(CH_2_)_3_NH_2_ and pure CA membranes, two plots were made on the same graph: (1) R as a function of the solute MW with the intersection of the horizontal line at R = 90%, and (2) a plot of the linearization of R, $\log(\frac{R}{1\;-\;R})$, as a function of solute MW with the intersection of the horizontal (dashed) line at $\log\left(\frac{R}{1\;-\;R}\right)=0.95$.

#### 2.5.3. Rejection Coefficients to Low-Molecular Weight Water-Soluble Uremic Toxins

The rejection coefficients, R, of the CA-SiO_2_-(CH_2_)_3_NH_2_ and pure CA membranes to markers for low-molecular weight water-soluble UTs: urea, creatinine, and uric acid, were determined by Equation (12). The initial feed solutions of each solute were prepared according to the highest reported concentrations of urea [38], creatinine [38], and uric acid [39]: 4.6 g/L, 240 mg/L, and 83 mg/L, respectively. The permeation studies were performed for 1.5 h at a QF of 100 ± 5 mL/min and TMP of 0.06 bar (44 ± 1 mmHg). Solute concentrations were determined in the permeate and feed samples using a UV-Vis spectrophotometer (UV-1700 PharmaSpec, Shimadzu, Japan), according to each toxin’s wavelength of maximum absorbance, λmax: 200, 230, and 293 nm for urea [40], creatinine [41], and uric acid [42], respectively.

#### 2.5.4. Bovine Serum Albumin Filtration

The rejection coefficients, R, of the CA-SiO_2_-(CH_2_)_3_NH_2_ and pure CA membranes to bovine serum albumin (BSA) were determined by Equation (8). The initial concentration of the feed solutions was 884 ± 4 mg/L, and the permeation studies were performed for 465 min at a Q_F_ of 100 ± 5 mL/min and TMP of 0.07 bar (52 ± 5 mmHg). Solute concentrations were determined in the permeate, initial feed, and final feed samples through the Bradford assay using a UV-Vis spectrophotometer (UV-1700 PharmaSpec, Shimadzu, Japan). Specifically, the absorbance of feed samples was measured at 595 nm, following the Bradford assay [43], whereas the absorbance of permeate samples was simultaneously measured at 590 nm and 450 nm according to a modified Bradford assay [44], allowing the determination of lower BSA concentrations.

## 3. Results and Discussion

### 3.1. Membrane Chacracterization

#### 3.1.1. Scanning Electron Microscopy (SEM)

**Figure 4:**
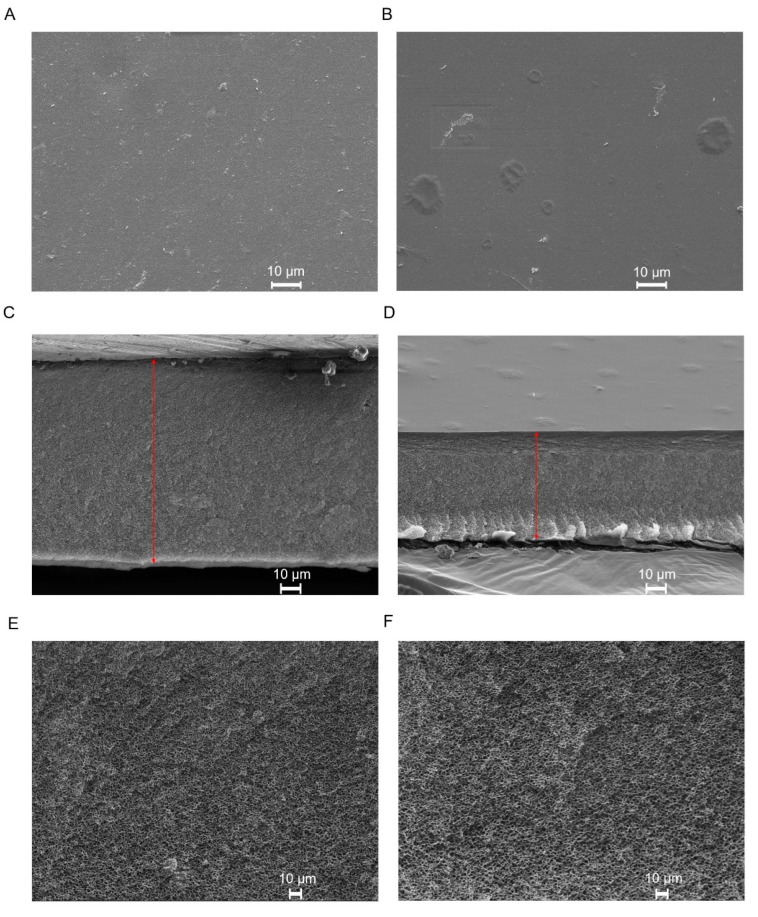
SEM images of the CA-SiO_2_-(CH_2_)_3_NH_2_ (**A**,**C**,**E**) and CA (**B**,**D**,**F**) membranes. (**A**,**B**): top active layer (1000×). (**C**,**D**): cross-section (700×) where the red lines indicate the membranes’ thickness. (**E**,**F**): bottom porous surface (4000×).

Figure 4 shows the SEM micrographs obtained for the active layer surface, cross-section, and bottom porous surface of the dried CA-SiO_2_-(CH_2_)_3_NH_2_ and CA membranes. The active layer of the membranes (Figure 4A,B) is responsible for the membrane selectivity, and both membranes exhibited completely dense active layers with no visible pores at a magnification of 1000×. The cross-section images (Figure 4C,D) confirmed the presence of a very thin skin layer of <1 μm for both membranes, outlining a much thicker, porous substructure. The overall thickness of the membranes was obtained from the cross-section images, yielding 103 ± 1 μm and 53 ± 1 μm for the CA-SiO_2_-(CH_2_)_3_NH_2_ and CA membranes, respectively. These results suggest that the introduction of silanol groups from the inorganic (TEOS) and hybrid (APTES) monomers led to membranes with increased thickness when compared to pristine CA membranes. This is in accordance with what has been observed in previous studies [25,27]. The porous structure (Figure 4E,F) confers mechanical strength to the monophasic hybrid membranes, while offering little or no resistance to the permeation of water and solutes. The average pore diameter in the porous surface for the monophasic hybrid and pristine CA membranes was 113 nm and 84 nm, respectively.

#### 3.1.2. Attenuated Total Reflection-Fourier Transform Infrared Spectroscopy (ATR-FTIR)

**Figure 5:**
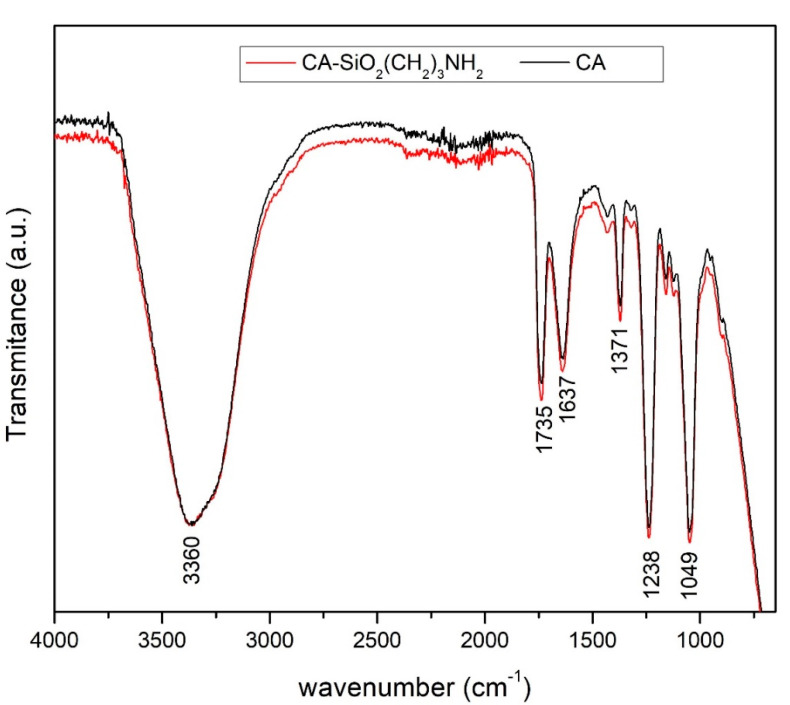
Wide-range ATR-FTIR spectra (4000–650 cm^−1^) of the CA-SiO_2_-(CH_2_)_3_NH_2_ and CA membranes.

The ATR-FTIR spectra of the CA-SiO_2_-(CH_2_)_3_NH_2_ and CA membranes’ active layer are compared in Figure 5.

The broad band centered at ~3360 cm^−1^ was assigned to the OH stretching mode, ν(OH), and contained contributions from the hydroxyl groups of non-esterified cellulose, as well as from adsorbed water and hydrolyzed silica precursors (TEOS and APTES). Moreover, the bending fundamental, δ(OH), for the adsorbed water was located at ~1637 cm^−1^, which has been previously described by other authors [45].

Similar to a previous work, [46], the strong carbonyl stretching mode, ν(C=O), appeared at 1735 cm^−1^, while the antisymmetric, ν_as_(C–O–C), and symmetric, ν_s_(C–O–C), stretching modes of the ester appeared as strong bands at 1238 cm^−1^ and 1049 cm^−1^, respectively.

There were no distinguishable CH stretching bands of cellulose acetate, which were possibly hindered by the broad band assigned to adsorbed water. However, in the CH deformation region, there was a medium band, typical of the methyl umbrella mode of acetate groups, at 1371 cm^−1^.

Amine functionalization of CA-SiO_2_-(CH_2_)_3_NH_2_ membranes with the same composition has been previously proven [27], despite there being no evident band of the NH bond in Figure 5. In fact, primary aliphatic amine stretching mode was assigned in the region of 3330–3340 cm^−1^, for which the band may have overlapped with the broad band which was assigned to ν(OH) [47].

Two important bands attributed to SiO_2_, namely ν(Si–O–Si) and ν(Si–O–C), occured in the regions of 1055–1165 cm^−1^ and 1115–1175 cm^−1^, respectively. Similar to previous studies [24], and due to the low silica content in the CA-SiO_2_-(CH_2_)_3_NH_2_ membrane, these bands were overlaid by those attributed to the ester stretching modes.

### 3.2. Characterization of the SHDMM: Pressure Profile, Pressure Drop, Transmembrane Pressure, Microchannel Height, Shear Rate and Shear Stress at the Wall

**Figure 6:**
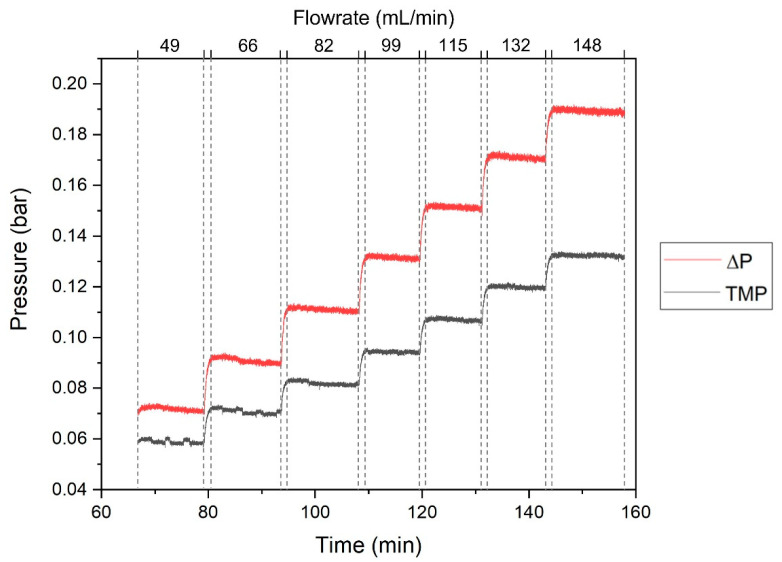
ΔP (red line) and TMP (black line) values obtained for the flow of DI water through the SHDMM circuit at different values of Q_F_, which are delimited by the dashed lines.

Figure 6 shows the values of ΔP and TMP obtained when DI water was circulated through the SHDMM containing the CA-SiO_2_-(CH_2_)_3_NH_2_ membrane at different values of Q_F_. The values of ΔP were 0.072, 0.091, 0.11, 0.13, 0.15, 0.17, 0.19 bar (54, 68, 83, 99, 114, 128, and 142 mmHg) for Q_F_ values of 49, 66, 82, 99, 115, 132, and 148 mL/min, respectively. The values of TMP were 0.059, 0.071, 0.082, 0.094, 0.11, 0.12, and 0.13 bar (45, 53, 61, 71, 80, 90, and 100 mmHg) for QF values of 49, 66, 82, 99, 115, 132, and 148 mL/min, respectively.

Typical values of Q_F_ under which commercial hemodialysis equipment operates range from 250 to 400 mL/min [48,49] and TMP should not exceed 0.4 bar (300 mmHg), according to the European Renal Best Practice (ERBP) [50]—with the latter being in the same range as the operating conditions of the SHDMM circuit. The Q_F_ is, however, below the typical values of hemodialysis equipment in order to avoid high values of sheer rate, as described below.

Considering the viscosity of water at 37 °C, μ_water_ = 0.6913 mPa.s [51], the height of the feed microchannel, calculated by Equation (3), was 205 ± 3 µm and 255 ± 10 μm when the CA-SiO_2_-(CH_2_)_3_NH_2_ and CA membranes were placed inside the SHDMM, respectively. The difference in channel height of the SHDMM feed channel when it encased the CA membrane was expected, as the total thickness of this membrane was approximately 50 µm lower than the thickness of the CA-SiO_2_-(CH_2_)_3_NH_2_ membrane.

The shear rate for each of the Q_F_ values was calculated by Equation (5) and was found to be 3887, 5235, 6504, 7852, 9122, 10,470, and 11,739 s^−1^ for Q_F_ values of 49, 66, 82, 99, 115, 132, and 148 mL/min, respectively. It is known that high fluid shear triggers the activation of platelets and their subsequent aggregation [52]. An in vitro study by Holme et al. showed that platelet activation and aggregation was observed at shear rates of 10,500 s^−1^ and above [53]. Therefore, it is safe to use all the tested feed flow rates up until 115 mL/min.

### 3.3. Permeation Experiments under Dynamic Conditions

#### 3.3.1. Water Permeability

**Figure 7:**
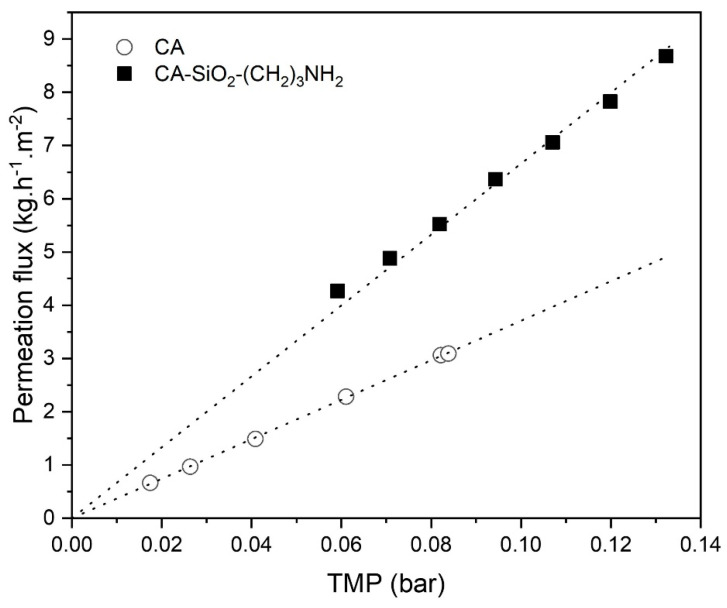
Ultrafiltration flux of pure water at 37 °C as a function of the transmembrane pressure for CA-SiO_2_-(CH_2_)_3_NH_2_ (■) and pure CA (○) membranes. The dotted lines represent the linear fit, which enables the determination of each membrane Lp.

Figure 7 shows the results of ultrafiltration flux, J, as a function of TMP obtained for the CA-SiO_2_-(CH_2_)_3_NH_2_ and pure CA membranes at 37 °C. The water permeability, L_P_, of the CA-SiO_2_-(CH_2_)_3_NH_2_ and pure CA membranes was 66.61 kg·h^−1^·m^−2^·bar^−1^ and 37.09 kg·h^−1^·m^−2^·bar^−1^, respectively. For the sake of standardization, the ultrafiltration fluxes of both membranes were corrected to 25 °C through Equations (8)–(11) and revealed L_P_ values of 50.77 kg·h^−1^·m^−2^·bar^−1^ and 28.27 kg·h^−1^·m^−2^·bar^−1^ for the CA-SiO_2_-(CH_2_)_3_NH_2_ and CA membranes, respectively. The introduction of silica followed by the presence of amine groups increased the hydraulic permeability of the monophasic hybrid membrane by a factor of 1.8 when compared to the pure CA membrane.

Current hemodialysis equipment should deliver ultrafiltration rates between 10–13 mL/(h·kg) [54], under an operating TMP of between 0.133 and 0.200 bar (100 and 150 mmHg). Hence, for a 70 kg adult, the expected ultrafiltration rate in a clinical scenario should not be lower than 700 mL/h. Considering the estimated value of L_P_ for the CA-SiO_2_-(CH_2_)_3_NH_2_ membrane, 89.40 mL·h^−1^·m^−2^·mmHg^−1^, and for an average TMP of 0.167 bar (125 mmHg) in order to achieve the 700 mL/h threshold, a total membrane surface area of 0.06 m^2^ would be enough. This is a promising result, as the estimated surface area is well below the effective permeation area of typical hemodialyzers, which ranges from 0.8 to 2.2 m^2^ [55].

#### 3.3.2. Molecular Weight Cut-Off

**Figure 8:**
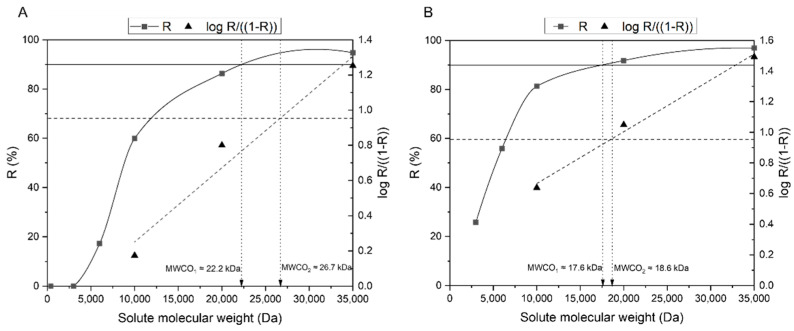
Rejection coefficient profiles of the CA-SiO_2_-(CH_2_)_3_NH_2_ (**A**) and CA (**B**) membranes to solutes with increasing molecular weights: 400, 3000, 6000, 10,000, 20,000, and 35,000 Da. The horizontal full lines are set for a rejection of 90%, i.e., $\log\left(\frac{R}{1\;-\;R}\right)=0.95$. The dashed rejection lines represent the linearization of the rejection coefficients obtained for 3 different molecular weight solutes: 10,000, 20,000, and 35,000 Da. The dotted dropdown lines indicate the estimated MWCO according to the method employed.

The MWCO was determined by two methods which are shown in Figure 8. The upper part of the curve resembled a plateau, hindering an accurate determination of the value corresponding to the intersection of the rejection coefficient curve with the horizontal rejection line (R = 90%). Hence, to overcome this ambiguity, the higher range of rejection coefficients was linearized, namely those corresponding to PEG 35,000, PEG 20,000, and PEG 10,000—enabling a precise determination of the intersection point.

The depicted values of MWCO corresponded to the intersection point of the plotted rejection coefficient curve (MWCO_1_) and subsequent linearization (MWCO_2_) with the respective horizontal rejection line. Hence, the MWCO was estimated to range between 22.2 and 26.7 kDa for the CA-SiO_2_-(CH_2_)_3_NH_2_ membrane, and between 17.6 and 18.6 kDa for the CA membrane. With the understanding that both membranes reject solutes with MWs greater than 20 kDa, it was predicted that vital blood components such as albumin, platelets, and blood cells would be rejected by both membranes. Furthermore, it is envisioned that molecules belonging to two different classes of uremic toxins proposed by the EUTox—small water-soluble compounds and middle molecules—can be removed, as they are able to cross the membrane. Prototypes to small water-soluble compounds and middle molecules include urea and β_2_-microglobulin, with a respective MW of 60 Da and 11,818 Da [2]. The third group of uremic toxins, PBUTs, remains a challenge: whilst the free fraction of these molecules (MW < 500 Da) is able to cross the membrane and successfully be removed from the blood circulation, the rejection of the bound fraction will be hindered by the large MW associated with the complexes they form with albumin (>60 kDa).

#### 3.3.3. Rejection Coefficients to Low-Molecular Weight Water-Soluble Uremic Toxins

**Figure 9:**
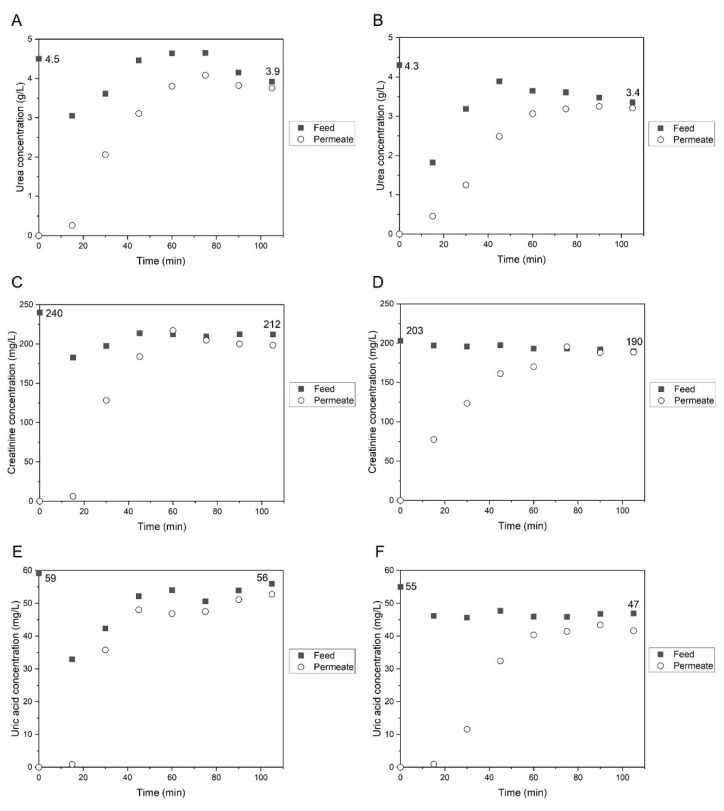
Concentration profiles of urea, creatinine, and uric acid for CA-SiO_2_-(CH_2_)_3_NH_2_ (**A**,**C**,**E**) and CA (**B**,**D**,**F**) membranes, regarding feed (■) and permeate (○) samples, for a total experiment time of 90 min. The initial feed concentrations aim to represent those of uremic populations.

Permeation experiments to prototype low-molecular weight water-soluble UTs (urea, creatinine, and uric acid) were performed with consideration to the highest reported concentrations found in uremic populations. Feed and permeate samples were collected at times of 0, 15, 30, 45, 60, 75, 90, and 105 min, with the respective concentrations represented in Figure 9.

The CA-SiO_2_-(CH_2_)_3_NH_2_ and CA membranes exhibited a similar behavior in terms of permeation of urea, creatinine, and uric acid. The initial concentrations of the feed solutions were defined according to the highest reported concentrations found in ESRD patients for the three uremic toxins [38,39], and these were measured after they had been prepared and before they were fed into the SHDMM system (t = 0 min). It should be noted that, while the highest reported uremic concentration for uric acid was 83 mg/L, the solubility limit of this toxin in water, at 20 °C, is 60 mg/L.

Prior to each experiment, the SHDMM system was primed with water so that there are no air bubbles present in the system, as well as to prevent the CA and CA-SiO_2_-(CH_2_)_3_NH_2_ membranes from becoming dry. The priming volume of the system was approximately 130 mL and, when the initial feed solution containing each of the three toxins was circulated into the SHDMM system, a dilution occurs. This explains the decrease in the initial concentration of the primary feed solution at t = 0 min (measured after it was prepared and before it was fed into the SHDMM system) and the moment it was collected after 15 min of circulation in the system (t = 15 min).

In general, for both the CA and CA-SiO_2_-(CH_2_)_3_NH_2_ membranes, it was observed that, between 15 and approximately 50 min, the concentration of the solutes in the feed compartment increased, and this could be explained by the decrease of water in the system, which was constantly being removed by convection from the feed compartment through the membrane and into the permeate channel. Between ~50 and 105 min, the concentration of the feed solution tended to stabilize at values close to the ones of the initial feed solution. To make this clear, the values of the initial and final concentrations of the feed solution are shown in each graph of Figure 9.

Regarding the concentration of the permeate solution, at the beginning of the experiment (t = 0), the collecting permeate chamber was filled with water, and as expected, the solute concentration in the permeate chamber was 0. The concentrations of the permeate solution increased considerably between 0 and approximately 50 min and, towards the end of the experiment (t > 80 min), they approached concentration values similar to those of the feed solution (at the corresponding time). This behaviour clearly demonstrates that the membranes are permeable to the three low-molecular weight water-soluble uremic toxins evaluated—creatinine, uric acid, and urea.

The rejection coefficients (Equation (8)) were calculated after 90 min of permeation. The rejection coefficient of the CA-SiO_2_-(CH_2_)_3_NH_2_ and CA membranes towards uric acid was 6% and 11%, respectively, while both membranes had a rejection factor of 4% towards urea. The rejection coefficient of the CA-SiO_2_-(CH_2_)_3_NH_2_ and CA membranes towards creatinine was 7% and 1%, respectively. These results are in agreement with what was discussed before in terms of MWCO, given that urea (MW 60 Da), creatinine (113 Da), and uric acid (168 Da) have much lower MWs than the CA-SiO_2_-(CH_2_)_3_NH_2_ and CA membranes’ MWCO.

#### 3.3.4. Bovine Serum Albumin Filtration

**Figure 10:**
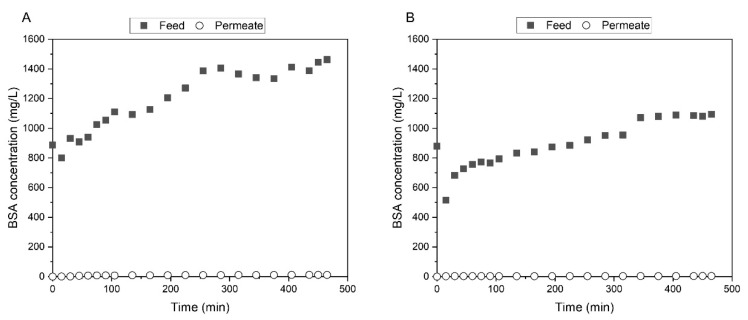
BSA concentration profiles for CA-SiO_2_-(CH_2_)_3_NH_2_ (**A**) and CA (**B**) membranes, regarding feed (■) and permeate (○) samples, for a total experiment time of 465 min.

Figure 10 shows the concentration profiles of BSA in the feed and permeate channels of the SHDMM equipped with the CA-SiO_2_-(CH_2_)_3_NH_2_ (Figure 10A) and CA (Figure 10B) throughout the recirculation experiments lasting 465 min. The aim of this experiment was to determine the rejection coefficients of the CA-SiO_2_-(CH_2_)_3_NH_2_ and CA membranes towards BSA and to assess the potential fouling of the membrane, mostly attributed to irreversible protein deposition and pore blockage.

As was described for the UT permeation experiments (Section 3.3.3), prior to initiating the filtration of the BSA solution with initial concentrations of 884 ± 4 mg/L, the SHDMM system was primed with water. The total priming volume for the permeation experiment with the CA membrane was approximately 130 mL and for the experiment with the CA-SiO_2_-(CH_2_)_3_NH_2_ membrane, it was reduced to 50 mL. This explains the decrease in concentration of the initial feed solutions of BSA between 0 and 15 min. From 15 to 465 min, the concentration of BSA in the feed solution of both permeation experiments through the CA and CA-SiO_2_-(CH_2_)_3_NH_2_ membranes increased steadily. This gives a clear indication that both membranes fully rejected BSA.

At the beginning of each experiment (t = 0 min), the permeate chamber was filled with water and therefore the permeate concentration was 0 mg/L. The permeation experiments were carried out for over 7 h and, throughout the entire experiment, the highest concentration of BSA detected in the permeate was 12 mg/L.

Furthermore, there seems to be no clear evidence of fouling events, as both the TMP and the ΔP remained approximately constant throughout the experiment, at 0.076 bar (57 ± 7 mmHg) and 0.12 bar (87 ± 3 mmHg) for the CA-SiO_2_-(CH_2_)_3_NH_2_ membrane, and 0.063 bar (47 ± 3 mmHg) and 0.077 bar (58 ± 2 mmHg) for the CA membrane, respectively. The ultrafiltration rate did not change significantly for any of the two membranes throughout the long-term filtration experiment, adding more evidence to support the absence of membrane fouling. At the end of the experiment, the heights of the feed microchannel of the SHDMM were 215 μm and 246 μm, when the SHDMM encased the CA-SiO_2_-(CH_2_)_3_NH_2_ and CA membranes, respectively. These values are very similar to those found using pure water at a Q_F_ of 100 mL/min (Section 3.2): 205 μm and 255 μm, for the SHDMM with the CA-SiO_2_-(CH_2_)_3_NH_2_ and CA membranes, respectively. This indicates that the protein deposition and adhesion is negligible, as there is no evident narrowing of the microchannel height even after 465 min of BSA filtration.

The rejection coefficient towards BSA was calculated with the BSA concentrations measured at the end of the experiment (after 465 min of filtration) and was 99% for both the CA-SiO_2_-(CH_2_)_3_NH_2_ and CA membranes. These results were expected given that BSA has a MW of 66.5 kDa which is similar to human serum albumin (HSA) [56], and the MWCO of the CA-SiO_2_-(CH_2_)_3_NH_2_ and CA membranes is approximately 27 kDa and 19 kDa, respectively.

## 4. Conclusions

A novel integrally skinned cellulose acetate-based monophasic hybrid amine-functionalized membrane, CA-SiO_2_-(CH_2_)_3_NH_2_, was synthesized by an innovative method which combines the phase inversion and sol-gel techniques.

SEM micrographs of the CA-SiO_2_-(CH_2_)_3_NH_2_ membrane cross-sections confirmed the existence of a thin dense active layer and a much thicker, porous layer. SEM analysis revealed that the total thickness of the CA-SiO_2_-(CH_2_)_3_NH_2_ membrane—103 μm—was approximately two times higher than the thickness of the CA membrane—54 μm.

Permeation studies showed enhanced mass transfer properties for the monophasic hybrid membrane when compared to the pure CA membrane, as evidenced by the increase of hydraulic permeability, measured at 37°C, from 37.09 kg·h^−1^·m^−2^·bar^−1^ for the CA membrane, to 66.61 kg·h^−1^·m^−2^·bar^−1^ for the CA-SiO_2_-(CH_2_)_3_NH_2_ membrane. Furthermore, the MWCO also increased from 18.1 kDa for the CA membrane, to 24.5 kDa for the CA-SiO_2_-(CH_2_)_3_NH_2_ membrane. Both membranes assured the permeation of water-soluble toxins such as urea, creatinine, and uric acid. Long-term continuous studies of BSA filtration revealed that both membranes completely reject albumin and that there is no evidence of irreversible fouling or albumin leakage in the SHDMM.

## Data Availability

Not applicable.
